# Predicting Successful Treatment Outcome of Web-Based Self-help for Problem Drinkers: Secondary Analysis From a Randomized Controlled Trial

**DOI:** 10.2196/jmir.1102

**Published:** 2008-11-22

**Authors:** Heleen Riper, Jeannet Kramer, Max Keuken, Filip Smit, Gerard Schippers, Pim Cuijpers

**Affiliations:** ^4^Amsterdam Institute for Addiction ResearchAcademic Medical CentreUniversity of AmsterdamAmsterdamThe Netherlands; ^3^Cognitive Science Centre AmsterdamUniversity of AmsterdamAmsterdamThe Netherlands; ^2^Department of Clinical Psychology and EMGO InstituteVU University Medical CentreAmsterdamThe Netherlands; ^1^Innovation Centre of Mental Health and TechnologyTrimbos Institute (Netherlands Institute of Mental Health and Addiction)UtrechtThe Netherlands

**Keywords:** Predictors, drinking, problem drinking, attribute-treatment interaction, Web-based intervention, self-help, pragmatic randomized trial, alcohol, general population

## Abstract

**Background:**

Web-based self-help interventions for problem drinking are coming of age. They have shown promising results in terms of cost-effectiveness, and they offer opportunities to reach out on a broad scale to problem drinkers. The question now is whether certain groups of problem drinkers benefit more from such Web-based interventions than others.

**Objective:**

We sought to identify baseline, client-related predictors of the effectiveness of Drinking Less, a 24/7, free-access, interactive, Web-based self-help intervention without therapist guidance for problem drinkers who want to reduce their alcohol consumption. The intervention is based on cognitive-behavioral and self-control principles.

**Methods:**

We conducted secondary analysis of data from a pragmatic randomized trial with follow-up at 6 and 12 months. Participants (N = 261) were adult problem drinkers in the Dutch general population with a weekly alcohol consumption above 210 g of ethanol for men or 140 g for women, or consumption of at least 60 g (men) or 40 g (women) one or more days a week over the past 3 months. Six baseline participant characteristics were designated as putative predictors of treatment response: (1) gender, (2) education, (3) Internet use competence (sociodemographics), (4) mean weekly alcohol consumption, (5) prior professional help for alcohol problems (level of problem drinking), and (6) participants’ expectancies of Web-based interventions for problem drinking. Intention-to-treat (ITT) analyses, using last-observation-carried-forward (LOCF) data, and regression imputation (RI) were performed to deal with loss to follow-up. Statistical tests for interaction terms were conducted and linear regression analysis was performed to investigate whether the participants’ characteristics as measured at baseline predicted positive treatment responses at 6- and 12-month follow-ups.

**Results:**

At 6 months, prior help for alcohol problems predicted a small, marginally significant positive treatment outcome in the RI model only (beta = .18, *P* = .05, *R^2^* = .11). At 12 months, females displayed modest predictive power in both imputation models (LOCF: beta = .22, *P* = .045, *R^2^* = .02; regression: beta = .27, *P* = .01, *R^2^* = .03). Those with higher levels of education exhibited modest predictive power in the LOCF model only (beta = .33, *P* = .01, *R^2^* = .03).

**Conclusions:**

Although female and more highly educated users appeared slightly more likely to derive benefit from the Drinking Less intervention, none of the baseline characteristics we studied persuasively predicted a favorable treatment outcome. The Web-based intervention therefore seems well suited for a heterogeneous group of problem drinkers and could hence be offered as a first-step treatment in a stepped-care approach directed at problem drinkers in the general population.

**Trial Registration:**

International Standard Randomized Controlled Trial Number (ISRCTN): 47285230; http://www.controlled-trials.com/isrctn47285230 (Archived by WebCite at http://www.webcitation.org/5cSR2sMkp).

## Introduction

Problematic alcohol use is not only a pervasive individual problem; it also imposes serious health and social burdens on the general population [1,2,3]. This makes it a major public health concern. Brief interventions offer the promise of easing these burdens, and their cost-effectiveness has been amply demonstrated in a number of studies and meta-analyses [4-9]. Yet in view of the small-to-medium treatment effects that have been reported by meta-analyses [4,6], it appears that not every problem drinker benefits equally from brief interventions. Web-based self-help interventions for problem drinking are the newest branch in the tree of brief interventions making it possible to reach out to problem drinkers on a broad scale at a relatively low cost. These Web-based interventions are clearly coming of age for a number of psychological disorders [10,11] and increasingly for alcohol problems as well [12,13]. As yet, however, the effect sizes found for brief Web-based interventions for problem drinking have not differed much from those for offline brief interventions [12,14]. The question therefore arises whether such Web-based interventions might work more effectively for some people than for others. The answer to this question could help to improve intervention development, treatment outcomes, and the matching of clients to treatment modalities, and is therefore of potential clinical, social, and economic interest [3,15].

It is well known that treatment response is not influenced by treatment alone [16]. A number of effect moderators of alcohol treatment outcomes have been identified [17]. These include clients’ baseline sociodemographics, within-treatment variables such as treatment fidelity, and posttreatment factors like social support for curbing drinking activities [18]. Prediction studies have provided a limited number of consistently identified baseline predictors of treatment outcome, including readiness to change problematic alcohol use [19,20,21], self-efficacy [19,20,22], and severity of alcohol use [4,16]. The milestone study by Project MATCH [19] is the best known example. Most prediction studies, however, have focused on severely alcohol-dependent clinical populations, and far fewer have focused on brief interventions for clinical populations in primary care settings or on problem drinkers in the general population [16,19]. Research suggests that baseline characteristics are more likely to affect treatment outcomes for less severe problem drinkers than for more highly dependent clinical populations [23].

We therefore investigate here whether specific baseline characteristics can be identified as predictors of a positive treatment outcome for problem drinkers in the Dutch population who completed a Web-based self-help intervention called Drinking Less. On the basis of predictors already reported in the literature, we hypothesized that six putative baseline characteristics—(1) female gender, (2) higher education, (3) Internet competence, (4) a moderate level of problem drinking, (5) prior professional help for problem drinking, and (6) high expectancy for positive results from a Web-based intervention—would interact with Drinking Less to predict a more favorable treatment outcome at follow-up. We conducted a secondary analysis of our Drinking Less trial data [14] to examine attribute-treatment interaction (ATI)—the interplay between the baseline characteristics (attributes) of problem drinkers and the intervention itself—and the influence such interaction might have on treatment response [24]. Drinking Less has been shown effective for problem drinkers who want to reduce their alcohol intake, yielding a medium effect size at 6-month follow-up (*d* = 0.40, 95% CI 5.86 - 18.10; *P* < .001). At 12 months, the difference between the groups had faded (*d* = 0.01, 95% CI -2.63 ~ 9.20, *P* = .21), mainly due to a further decrease in alcohol consumption in the control group. Results of this pragmatic randomized trial have been reported elsewhere [14].

To the best of our knowledge, this is the first article that uses randomized trial data to assess predictors of short- and longer-term outcomes in Web-based self-help for problem drinkers in the general population.

## Methods

### Participants and Procedure

Data were retrieved from a pragmatic randomized trial with two parallel groups using block randomization stratified for gender, with follow-up at 6- and 12 months [14]. In brief, we recruited adult participants from the general population through advertisements in national newspapers and health-related websites. The study and intervention were conducted entirely via the Internet, with the exception of the informed consent form which had to be signed and returned by post. In the inclusion criteria, we applied different cut-off points for problem-drinking men and women. Men were selected who were drinking either more than 21 standard units per week (excessive drinking) or 6 or more units at least 1 day per week for the past 3 months (hazardous drinking). Women were included if they drank over 14 units a week or 4 or more units at least 1 day a week for the past 3 months. One standard unit represents 10 g of ethanol. Additional inclusion criteria were: age 18-65, access to the Internet, and no previous professional help for problem drinking at the start of the study.

**Figure 1:**
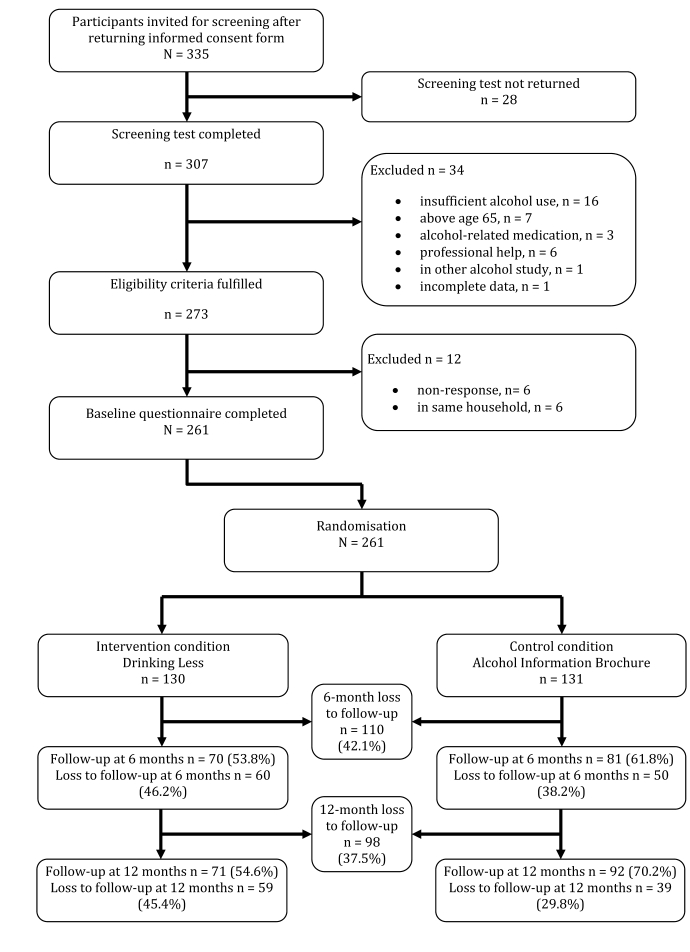
Flow of participants through the trial

We kept our exclusion criteria to a minimum to facilitate a low-threshold inclusion strategy consistent with the nature of self-help interventions without therapeutic guidance. We therefore did not conduct diagnostic interviews. After screening and baseline assessment, participants were randomly assigned to the experimental condition (the Drinking Less intervention) or to the control condition (an online psychoeducational brochure on alcohol use that could be read in 10 minutes) [25]. We selected a total of 261 adult problem drinkers. Figure 1 shows the flow of participants through the trial.

### Intervention

**Figure 2:**
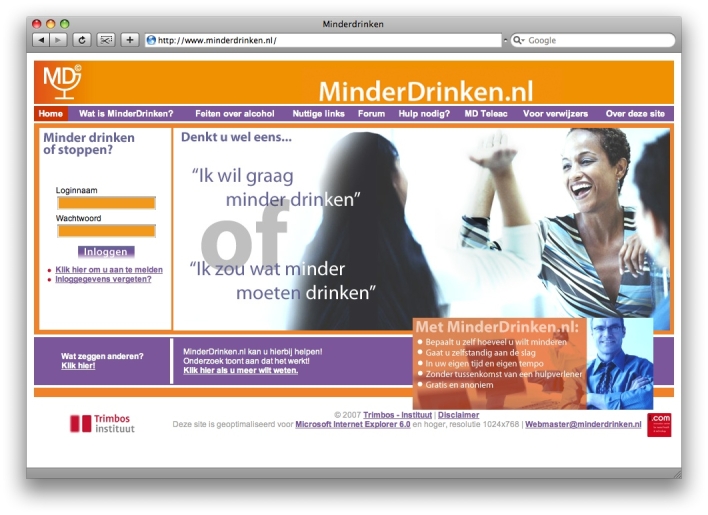
Drinking Less home page [26]

Participants in the experimental condition received access to the Drinking Less intervention [26]. Drinking Less is a free-access, Web-based self-help intervention without therapist guidance for problem drinkers who want to reduce their alcohol consumption, preferably to within the recommended Dutch limits for low-risk drinking [27]. The intervention is based on cognitive-behavioral and self-control principles [28,29] which are suitable for Web-based implementation due to their standardized nature and systematic approach. Drinking Less consists of a home page giving information on alcohol and treatment services and offering access to the self-help program via an automated sign-up procedure with a description indicating for whom the intervention is suitable (Figure 2). The program proceeds in four successive stages: (1) preparing for action; (2) goal setting; (3) behavioral change; and (4) maintenance of gains and relapse prevention. These stages contain elements known to be effective, such as goal setting and analysis of drinking behavior [29,30]. The self-help program also includes access to a moderated peer-to-peer discussion forum. The recommended treatment period is 6 weeks, which should give a reduction in alcohol consumption enough time to take hold [31]. Trial participants were allowed to use the intervention as long as they felt necessary. Access to Drinking Less proceeded through a unique log-in and security identification code and was available on a 24-hours-a-day, 7-days-a-week basis.

### Predictive Variables

Our choice of baseline participant characteristics as putative predictors was based on theoretical assumptions and results from previous prediction studies [16-22]. We selected six characteristics: (1) gender, (2) education, (3) Internet use competence (sociodemographic factors), (4) mean weekly alcohol consumption, (5) prior professional help for alcohol problems (level of problem drinking), and (6) participants’ expectancies of Web-based intervention as helpful for overcoming problem drinking.

### Outcome Measure

The outcome measure was defined as the individual differences between baseline (T0) mean weekly alcohol consumption and the mean level of consumption at posttreatment (6 months, T1) and at follow-up (12 months, T2) in the total group. Alcohol consumption was assessed with the Dutch version of Weekly Recall (WR) [32,33]. It records the number of units consumed in the 7 days preceding the assessment.

### Statistical Analyses

We first used *t*-tests, chi-square tests, and logistic regression to assess whether the randomization had resulted in two comparable groups at baseline and whether any differential loss to follow-up had occurred. We then performed intention-to-treat (ITT) analysis, using last-observation-carried-forward (LOCF) data and regression imputation (RI) to deal with loss to follow-up. Overall loss to follow-up was high (Figure 1), and we wanted to avoid overestimating the impact of the intervention [34]. ITT analysis enabled us to maintain sufficient power and the integrity of randomization. The LOCF imputation procedure assumes that outcome assessments of participants not reached for follow-up would equal their last available assessment [34]. Missing WR data at 6 months and 12 months were also estimated by RI, using the significant predictors for the missing outcomes and for dropout [34]. At 6 months those predictors were condition, baseline partner status, and baseline weekly alcohol units according to WR; at 12 months they were condition, gender, weekly alcohol units according to WR at 6 months (imputed), and baseline alcohol units as measured by the Dutch version of the Quantity-Frequency Variability Index (QFV) [35].

In the third step, we created dichotomous measures for the continuous and categorical baseline variables, alongside the already dichotomous variable of gender (female gender: yes/no). Values on the WR scale were transformed into a variable distinguishing moderate problem drinking (14 - 35 mean weekly alcohol units for women, 21 - 50 for men) from severe problem drinking (> 35 or > 50 units women/men). Categorical variables with more than two categories were recoded into two meaningful categories: (1) education: high/low (university and professional degrees versus the rest); (2) Internet competence: experienced/beginner; (3) prior professional help for alcohol problems: yes/no; and (4) expectancies of Web-based intervention: high/low. We then applied regression analyses to ascertain whether these particular groups benefited more from the intervention than others. We assessed the interactions between the above-baseline attributes and the Drinking Less intervention modality, and then the effects of those interactions on treatment outcome. In this model, the standardized individual change scores (pre- to post-intervention effect sizes) served as the dependent or outcome variable. The interaction terms of each participant characteristic with the intervention dummy (Drinking Less experimental condition = 1, control condition = 0) served as independent predictor variables, along with their constituent main effects.

We next calculated the product of the intervention dummy and each of the dummy variables describing the participants’ characteristics [36,37]. The interaction terms were entered together with the corresponding main effects into the linear regression model and tested at *P* < .05. Independent-samples *t*-tests were used to analyze differences between the conditions in terms of problem drinking outcome at T1 and T2. This technique permitted us to test for the differential effects of the predictors in interaction with the Drinking Less treatment. It also enhanced the power to detect effects. If neither of these interaction terms proved significant, then the effect of the predictor was deemed not to be modified by Drinking Less. That is, the effect of Drinking Less on drinking outcome could not be explained by the predictor’s modifying effect on the relationship between treatment and outcome.

We subsequently repeated this procedure in completers-only analyses on those participants who completed the follow-up questionnaire at 6 months (n = 151) or at 12 months (n = 163) to verify whether the results of the two ITT analyses would be sustained. Finally, we used descriptive statistics to illustrate the changes in alcohol consumption over time in terms of the identified predictors. The sample size provided 24 participants per variable at 6 months and 26 per variable at 12 months [38]. All analyses were conducted with SPSS version 15 and were carried out independently by two researchers to cross-check outcomes.

## Results

### Sample Characteristics

The demographic and clinical characteristics of participants at baseline are shown in Table 1. No differences were found between the experimental and control groups on any of these variables at baseline (even when tested conservatively at *P* < .10 to ensure against marginal differences that could affect results). This indicated that the randomization was successful. At baseline, all 261 participants (100%) were exceeding the mean number of weekly alcohol units set by the Dutch guideline for sensible drinking for healthy adults. Mean weekly alcohol intake was 43.6 standard units (SD = 21.6). More than half the sample belonged to the category of moderate, as opposed to severe, problem drinkers (n = 148, 57.7%). The female-to-male ratio was almost 1:1. Two-thirds of participants had high educational backgrounds (n = 182, 69.7%). Most participants considered themselves experienced Internet users (n = 204, 78.1%). Almost half had positive expectations of the intervention (n = 127, 48.2%). The large majority of participants (n = 231, 88.5%) were in the contemplation stage of change, meaning that they wanted to reduce their alcohol consumption in the near future [39,40]. Most (n = 243, 93.1%) aimed for moderation rather than abstinence. Few (n = 33, 12.6%) had ever received professional help for their problem drinking.

**Table 1:** Baseline characteristics of the 261 participants (values are numbers and percentages of participants, unless otherwise indicated)

	Condition^a^	
	Experimental n = 130	Control n = 131
Female gender^b^	64 (49.2)	64 (48.9)
Education^b^		
Low	41 (31.5)	38 (29.0)
High (academic/professional)^b^	89 (68.5)	93 (71.0)
High Internet competence^b^	104 (80.0)	100 (76.3)
High treatment expectancy^b^	61 (46.9)	66 (49.6)
Weekly alcohol intake in standard units^c^ (mean, SD)	43.7 (21.0)	43.5 (22.3)
Moderate problem drinking^b^ 14-35 units per week (women) 21-50 units per week (men)	74 (56.9)	74 (56.5)
Severe problem drinking > 35 (women) and > 50 (men) units^c^ per week	56 (43.1)	57 (43.5)
Prior professional help for problem drinking^b^	18 (13.8)	15 (11.5)
Contemplation stage^d^	116 (89.2)	115 (87.8)
Alcohol moderation as goal	120 (92.3)	123 (93.9)
Age (mean, SD)	45.9 (8.9)	46.2 (9.2)
Living with a partner	75 (57.7)	71 (54.2)
Paid employment	94 (72.3)	96 (73.3)

^a^All differences between conditions were non-significant (tested at *P* < .10).

^b^Indicates putative predictor of favorable treatment response.

^c^A standard unit contains 10 g of ethanol.

^d^Assessed with validated Dutch version of Readiness to Change Questionnaire [39].

### Predictors of Loss to Follow-up

Participants who did not return the questionnaire 6 months after baseline did not differ from posttreatment responders in terms of the characteristics assessed at baseline (*P* > .10; Table 1 for characteristics). Loss to follow-up at 6 months was 42.1% (n = 110) and was distributed rather evenly across the two conditions (n = 60 in the experimental and n = 50 in the control condition; χ^2^_1_ = 1.71, *P* = .19). At 12 months, loss to follow-up was 37% (n = 98) and was greater in the experimental condition (n = 59, 45% ) than in the control condition (n = 39, 30%; χ^2^_1_ = 5.56, *P* = .02). Non-responders at 12 months had a higher baseline mean weekly alcohol intake as measured by WR (46.9 units, SD = 24.3) than non-responders (41.7 units, SD = 19.7; *t*_259_ = 1.91, *P* = .06).

### Predictors of Successful Outcome: Mean Weekly Alcohol Consumption at 6 and 12 Months

Analyses of predictor-by-treatment interaction effects in terms of a successful reduction of mean weekly alcohol use at 6 and 12 months showed similar results for the last-observation-carried-forward (LOCF) and the completers-only model. We therefore present here only the intention-to-treat models. Results of the completers-only analysis are available from the first author.

Analyses of predictor-by-treatment interaction effects in terms of a successful reduction of mean weekly alcohol use found no significant effects for the putative predictors at 6 months (Table 2 and Table 3), with the exception of prior professional help for problem drinking, which emerged only after regression imputation (RI; Table 3). Its predictive power with regard to treatment response was only marginally significant and the explained variance was small (N = 261, beta .18, *P* = .05, *R^2^* = .11). At 12 months, female gender predicted successful alcohol reduction in both analysis models (Table 2 and Table 3). RI indicated a significant but small impact and explained variance (N = 261, beta = .27, *P* = .01, *R^2^* = .03), while the LOCF model showed a less strongly significant impact and a lesser amount of explained variance (N = 261, beta = .22, *P* = .045, *R^2^* = .02). High education level was identified as an additional predictor of successful outcome at 12 months. The LOCF analysis (N = 261, beta = .33, *P* = .01, *R^2^* = .03) showed a significant but modest effect and accounted for a small fraction of the variance in treatment outcome, but the effects in the RI model were not significant.

**Table 2:** Predictor-by-treatment interaction regressed individually using last-observation-carried-forward (LOCF) imputation at 6- and 12-month follow-up

Interaction term: participant characteristic by condition (Drinking Less = 1)	Effect on mean weekly alcohol consumption^a^ at 6 months (N = 261)			Effect on mean weekly alcohol consumption^a^ at 12 months (N = 261)		
	beta^b^	*P*	*R^2^*^c^	Beta^b^	*P*	*R^2^*^c^
Female	.003	.98	.03	.22	.045	.02
High educational level	.17	.17	.03	.33	.01	.03
High Internet competence	.13	.39	.03	.11	.44	.00
High treatment expectancy	.09	.37	.03	.09	.37	.00
Moderate problem drinking (female/male 14-35 or 21-50 units a week^a^)	-.02	.86	.03	.04	.70	.06
Prior help for drinking	.07	.48	.03	-.05	.60	.00

^a^measured in standard units containing 10 g of ethanol

^b^beta: standardized regression coefficient

^c^ *R^2^*: amount of variance in treatment response explained by the model

**Table 3:** Predictor-by-treatment interaction regressed individually using regression imputation (RI) at 6- and 12-month follow-up

Interaction term: participant characteristic by condition (Drinking Less = 1)	Effect on mean weekly alcohol consumption^a^ at 6 months (N = 261)			Effect on mean weekly alcohol consumption^a^ at 12 months (N = 261)		
	beta^b^	*P*	*R^2^*^c^	beta^b^	P	*R^2^*^c^
Female	.06	.53	.12	.27	.01	.03
High educational level	.11	.37	.10	.21	.10	.03
High Internet competence	.002	.99	.10	.06	.97	.01
High treatment expectancy	.15	.14	.11	.04	.74	.00
Moderate problem drinking (female/male 14-35 or 21-50 units a week^a^	-.08	.46	.16	-.09	.39	.17
Prior help for drinking	.18	.05	.11	.02	.79	.01

^a^measured in standard units containing 10 g of ethanol

^b^beta: standardized regression coefficient

^c^ *R^2^*: amount of variance in treatment response explained by the model

We compared the mean weekly alcohol consumption at 6 and 12 months for the two conditions as shown by the intention-to-treat and completers-only analyses. The last-observation-carried-forward (LOCF) model appeared to be the most conservative estimation method for the total group, as it returned the highest alcohol intake in both conditions—thus suggesting less improvement. We therefore chose these more cautious LOCF results to report outcomes for the two main predictors identified in our analysis. Detailed information about the other two models can be obtained from the first author.

**Figure 3:**
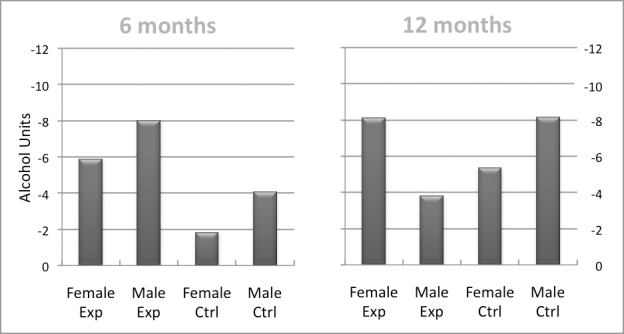
Reductions in mean weekly alcohol consumption (in mean weekly units containing 10 g of ethanol) in experimental and control groups 6 and 12 months after baseline, by gender (LOCF)

Figure 3 shows that women in the Drinking Less condition had not reduced their mean weekly alcohol consumption at 6 months to a greater degree than their male counterparts either in absolute terms (-5.86 vs -8.01 units) or in relative terms (-14.6% vs -16.9%). At 12 months, in contrast, women in the Drinking Less condition had reduced their intake (-8.13 units, -20.3% as compared to baseline) substantially more in both absolute and relative terms than female controls (-5.36 units, -15.3%) or than males in the experimental condition (-3.8 units, -8.0%). Interestingly, men in the control condition had decreased their intake at 12 months by a larger amount in absolute and relative terms (-8.16 units, -15.5%) than men who had completed the Drinking Less intervention (-3.8 units, -8.0%).

**Figure 4:**
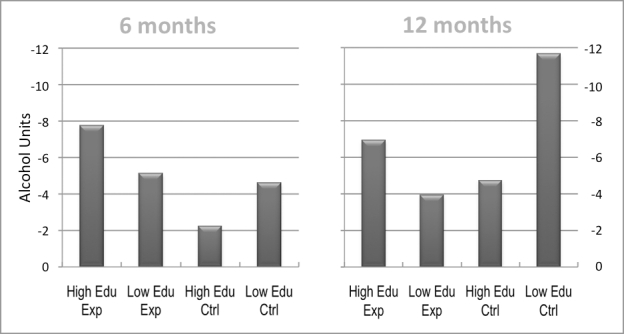
Reductions in mean weekly alcohol consumption (in mean weekly units containing 10 g of ethanol) in experimental and control groups 6 and 12 months after baseline, by high and low education (LOCF)

At 6 months, the more highly educated Drinking Less (experimental) participants had achieved the greatest reduction in both absolute and relative terms (-7.74 units, -19.0%) as compared to other categories (Figure 4). Although at 12 months their reduction had diminished by nearly one unit (0.80), they were still drinking less (-6.94 units, -17.1%) than at baseline, and their reduction remained greater than that of the lesser educated experimental participants (-3.93 units, -7.8%) and the more highly educated controls (-4.73, -11.6%). Interestingly, though, the lesser educated controls achieved the greatest reduction of all (-11.65 units, -23.1%) at 12 months.

## Discussion

The aim of this study was to determine whether some groups would benefit more than other groups from Drinking Less, a Web-based self-help intervention for problem drinkers, when assessed at 6 and 12 months. We investigated six characteristics of the participants at baseline as putative predictors of treatment response: (1) female gender, (2) high level of education, (3) high Internet experience, (4) moderate as opposed to severe level of problem drinking, (5) prior professional help for alcohol-related problems, and (6) high expectancies for Web-based intervention.

At the 6-month follow-up, we could not convincingly establish predictive value for any of these putative predictors, with the possible exception of prior help for alcohol problems, which was only marginally significant under the regression imputation model. Some other studies have likewise identified prior professional help as a predictor of positive client-by-treatment interaction leading to successful outcomes [23]. An explanation might be that reducing problem drinking requires multiple efforts over time (perhaps with a cumulative facilitating effect), and that help seeking is one such effort.

At 12 months, we found a modest prognostic value for female gender and for higher education; both variables were associated with better treatment response to the Drinking Less self-help intervention. Women who completed the intervention were found to have reduced their alcohol consumption to a significantly greater extent than men or than control group participants. Comparable results for female gender as a predictor of a successful brief intervention outcome in general population samples were reported by Sanchez-Craig and colleagues [31] and, to a lesser extent, for general practice patients by Reinhardt [41]. By contrast, several meta-analyses have found similar effectiveness of brief interventions for men and women in primary care populations [5,42] or even far stronger effects for men in general practice populations [9,43]. Women’s favorable results in our Web-based course for problem drinking are, however, in line with findings that e-health in general is of particular interest to women [44].

Higher levels of education also had modest predictive power and explained a small amount of variance at 12 months in combination with Drinking Less. This finding is consistent with results from other studies that identified high education as interacting with treatment interventions to produce favorable outcomes [18,45]. Like female gender, high education is also reportedly associated with a greater use of the Internet for health-related issues [46]. Interestingly, the added benefit of high education in the Drinking Less treatment outcome at 12 months coincided with a remarkable decrease in alcohol consumption by lesser educated male control group participants. On the basis of our data we can only hint at possible explanations, such as that our online psychoeducational information may have had a delayed but more effective long-term impact on men with lower levels of education. This issue needs further research.

The other characteristics investigated were not found to act as predictors in our study. A moderate baseline level of problem drinking (in terms of mean weekly alcohol consumption) did not predict better outcomes than a severe level. This contrasts with the many studies that assume brief interventions to be better suited to moderate problem drinkers [4]. One explanation could be the high level of motivation and readiness to change that we found in both moderate and severe drinkers in our self-referred study sample (Table 1). Another explanation could be that baseline severity of drinking is less relevant to treatment outcome for problem drinkers in the general population than for the more severely alcohol-dependent clinical samples that form the basis of many studies. The former group may be experiencing a range of incipient problems, such that their treatment response may be influenced by a wider range of factors, whereas the health and social problems of severely dependent drinkers may have already crystallized into more specific forms [23].

We did not find any predictive value for the two remaining putative predictors, Internet experience and positive expectancies of treatment efficacy, in contrast to some other studies that did [47,48]. Explanations might be that Drinking Less is equally suitable for both experienced and beginning Internet users and that positive expectations were what prompted both the experimental and control participants in our self-referred sample to take part in the first place.

### Limitations and Strengths

This study has several limitations that are important to acknowledge. We conducted secondary analysis of data from our pragmatic randomized trial [14].The overall loss to follow-up in that trial was substantial at both follow-up assessments (Figure 1). High dropout rates are common in self-help interventions for problem drinking without therapist guidance, whether Web-based or otherwise [49,50], but attrition rates appear to be especially high for those delivered over the Internet, as easy accessibility may also mean easy dropout.High loss to follow-up is therefore a potential concern in all Web-based self-help interventions [51,52].In the present study, we dealt with attrition data analytically as rigorously as possible by conducting intention-to-treat analyses, using last-observation-carried-forward and regression imputation. Nevertheless, the high loss to follow-up may still have biased our results by obscuring meaningful predictors.

Secondly, we conducted a prespecified subgroup analysis and hence cannot rule out false-positive or false-negative predictors resulting from multiple testing [53,54]. Given that we found only a marginally significant predictor (prior help) at 6 months and two further predictors (female gender and high educational level) at 12 months, this might well have been the case. On the other hand, we kept the number of putative predictors to a minimum and also appropriate in relation to our sample size [38]. The fact that we detected different predictors at 6- and 12-month follow-up could also mean that different factors operate at different points during the post-intervention period [16].

We were also limited by the data in the number of predictors we could investigate. That prevented us from studying self-efficacy, a potentially important predictor [21]. Nor could we investigate another key predictor, readiness to change [55], as most participants by far (n = 231, 88.5%; Table 1) were at the contemplation stage [39]. A final limitation is that our findings are generalisable only to self-referred problem drinkers in the general population who are motivated to take part in a Web-based self-help intervention.

Our study has a number of strengths as well. The study on which the analysis is based was one of the first pragmatic randomized trials on the effectiveness of Web-based self-help interventions without therapeutic guidance for problem drinkers in the general population. The data also enabled us to examine short- and longer-term relationships. Because we had anticipated a high overall loss to follow-up when we first selected the trial sample, we included enough participants to ensure the statistical power to detect differences between the experimental and control conditions and between subgroups [14].

### Conclusion

Female gender and a high level of education were found to have interacted with the Drinking Less self-help intervention to predict a somewhat better treatment response one year after the start of the intervention. This suggests that Web-based self-help without therapeutic guidance may hold a special attraction for problem drinkers with greater fears of stigmatization, including women or more highly educated people—population segments that might otherwise be difficult to reach with face-to-face brief interventions [56]. The non-stigmatizing approach to problem drinking in Web-based self-help and the lack of a need to interact with a therapist may form part of the appeal to these groups [44, 57]. 

At the same time, the effects of the predictors identified here offer only a very partial explanation for how client characteristics interact with treatment to affect outcome. Other baseline attributes such as self-efficacy may also play a role [21]. In addition, non-baseline predictors, including treatment progress factors (such as dose-response interaction stemming from variable treatment compliance) and posttreatment factors (such as social support), may prove to have stronger influences on client-by-treatment interaction and therapeutic outcomes, as has indeed been reported in clinical treatment samples [16,58].

### Implications for Public Health Strategies

Our findings could enhance public health strategies that use stepped-care approaches to curb problem drinking in the general population. Since none of the groups we identified stood out conspicuously against others as deriving benefit from Drinking Less, we would argue that Web-based self-help is well suited to a broad, heterogeneous group of problem drinkers. It may therefore serve well as an initial intervention in a stepped-care model, suitable for matching to a large and varied group of problem drinkers in the general population and not just at more individual levels [58,59]. The 24/7 free access to Drinking Less guarantees swift entry to the help program, and such ready access is known to facilitate positive outcomes as well as additional help-seeking behavior, if needed [60,61]. To sustain treatment progress, booster sessions might be needed 6 months after the intervention, in particular to support male participants.

### Future Studies

Our results add to the knowledge already gained from prediction studies in that we tested the role played by individual baseline attributes in the effectiveness of Web-based self-help for problem drinkers in the general population. The scope of future prediction research now needs to be extended to include the contributions of within-treatment progress variables, such as dose-response relationships and the time required to initiate positive behavioral change, and of posttreatment variables like social support. Replication of our study is needed in view of the novelty of Web-based interventions for problem drinkers and the related prediction research.

## Abbreviations

ATI: attribute treatment interaction
ITT: intention to treat
LOCF: last observation carried forward
QFV: quantity-frequency variability index
RI: regression imputation
WR: weekly recall
